# Analysis of dynamic acoustic resonance effects in a sonicated gas–liquid flow microreactor

**DOI:** 10.1016/j.ultsonch.2023.106300

**Published:** 2023-01-18

**Authors:** William Cailly, Keiran Mc Carogher, Holger Bolze, Jun Yin, Simon Kuhn

**Affiliations:** KU Leuven, Department of Chemical Engineering, Celestijnenlaan 200F, 3001 Leuven, Belgium

**Keywords:** Ultrasonic microreactors, Ultrasonic atomization, Nonlinear acoustics, Acoustic radiation force, Acoustic resonance

## Abstract

In this work, we characterize acoustic resonance phenomena occurring between gas bubbles in a segmented gas–liquid flow in a microchannel irradiated with a frequency around 500 kHz. A large acoustic amplitude can be reached, leading to gas–liquid interface deformation, atomization of micrometer sized droplets, and cavitation. A numerical approach combining an acoustic frequency-domain solver and a Lagrangian Surface-Evolver solver is introduced to predict the acoustic deformation of gas–liquid interfaces and the dynamic acoustic magnitude. The numerical approach and its assumptions were validated with experiments, for which a good agreement was observed. Therefore, this numerical approach allows to provide a description and an understanding of the acoustic nature of these phenomena. The acoustic pressure magnitude can reach hundreds of kPa to tens of MPa, and these values are consistent with the observation of atomization and cavitation in the experiments. Furthermore, volume of fluid simulations were performed to predict the atomization threshold, which was then related to acoustic resonance. It is found that dynamic acoustic resonance gives rise to atomization bursts at the gas bubble surface. The presented approach can be applied to more complex acoustic fields involving more complex channel geometries, vibration patterns, or two-phase flow patterns.

## Highlights

• Dynamic acoustic resonance in gas–liquid microreactors is modelled.
• Gas-liquid interface deformation and atomization are accurately predicted.
• Faraday instability at resonance peaks leads to transient atomization bursts.

## 1 Introduction

The integration of ultrasound actuation with micro-scale flow reactors is often chosen as a route to further intensify processes [1], [2], [3], [4], by *e.g.* improving mixing [5], [6], [7], and in case of two-phase flow interfacial mass transfer [8], [9]. In general, these ultrasonic microreactors are either operated in the low frequency (below 200 kHz) or high frequency (above 1 MHz) range. Low frequency ultrasound is associated with the formation of cavitation bubbles in the microchannels, which are then exploited to intensify mixing and interfacial mass transfer, but also for *e.g.* synergistic effects in particle synthesis [10], [11], [12], [13], [14]. Contrary to that, high frequency ultrasound is not dominated by cavitation effects, as the applied power levels do not exceed the cavitation threshold. However, the wavelength of the applied ultrasound in the fluid matches the channel size, making it possible to form a standing wave within the channel and utilize the associated effects, such as the acoustic radiation force and streaming [15], [16]. These effects are commonly exploited to manipulate particles [17], [18], [19], [20], [21], [22], and to enhance mixing [23], [24], [25], [26].

As highlighted by the examples above, the application of low and high frequency ultrasound in microreactors is quite advanced and has found various applications in biological, pharmaceutical and chemical processes. However, the transition frequency range (between 200 kHz and 1 MHz) is largely unexplored in microreactors. Recently, Mc Carogher et al. [27] reported the observation that gas–liquid segmented flow can act as a set of acoustic resonators in a frequency range from 100 kHz to 500 kHz. At resonance, a large acoustic amplitude is reached, leading to gas–liquid interface deformation, atomization of micrometer sized droplets, and cavitation. This resonance phenomenon in the transition frequency range could be exploited to *e.g.* increase interfacial area between the gas and liquid phase, and intensifying reactions that are limited either by mass transfer and/or phase distribution. However, so far no model to describe the acoustic phenomena and the acoustic behavior of the two phases in the microchannel is available. The development of such a model is paramount to increase the fundamental understanding of the resonance phenomenon, and to further design and optimize microreactors based on it.

In this work, a numerical modeling framework is presented which predicts the acoustic field in the microchannel, the deformation of the gas–liquid interface, and the atomization threshold. This approach focuses on modeling dynamic acoustic resonance, which is the main mechanism that induces the observed phenomenon. The next section briefly reviews the experiments used for validation, followed by the presentation of the model and its underlying assumptions. Then, the comparison between numerical results and experiments for different relevant configurations is presented, followed by an in-depth discussion and concluding remarks.

## 2 Experimental and numerical methods

### 2.1 Description of the experimental setup

Experiments were performed in two microreactor setups: (i) a silicone etched microreactor with 1.2 × 0.6 mm cross section square channel and (ii) a single glass channel with a 1.2 × 1.2 mm or 1.0 × 1.0 mm square cross section. Both microreactor setups are integrated with ultrasound by gluing a piezoelectric plate to the bottom surface, and the selected actuators were: (i) Pz26, 80 × 40 × 4.06 mm, (Ferroperm^TM^, Denmark), (ii) Pz26, 20 × 11 × 1.67 mm and 80 × 20 × 1.67 mm (Ferroperm^TM^, Denmark). A detailed description of the setups is given in Mc Carogher et al. [27]. Various gas–liquid flow rate ratios were delivered to the reactors to achieve gas–liquid segmented flow, while being sonicated at powers from 5 W to 20 W, and specific frequencies associated with actuator resonance from 40 to 600 kHz. The flow and the resulting acoustic resonance phenomena were recorded with a microscope (SMZ25, Nikon) equipped with a high-speed camera (Fastcam mini Ux100, Photron limited). To validate the model, the results for an actuation frequency of around 500 kHz were used, as the most significant phenomena were observed at this frequency. Long air bubbles (with a length of 2 to 6 times the channel height, typically used in practice) were considered. The liquid and gaseous stream are generated by equipment chosen to deliver a steady continuous volumetric stream without disruptions (Fusion 4000x Syringe pump, Chemyx Inc.^TM^, Stafford, Texas and EL-FLOW mass flow controller, Bronkhorst^TM^, Vorden NL). These steady streams are combined in a microfluidic junction built inside the chip. The steady conditions and microfluidic combination led to a repeatable and predictable process, so that each bubble was generated with the same volume after a defined time span. The relevant fluid properties and geometric parameters selected for this study are listed in Table 1.

**Table 1:** Fluid properties and geometric parameters implemented in the numerical model.

	Symbol	Value	Unit
Water			
Density	ρ	1000	kg/m^3^
Speed of sound	$c_{0}$	1480	m/s
Dynamic viscosity	μ	0.001	Pa⋅s
			
Air–Water			
Surface tension	γ	0.072	N/m
			
Configuration			
Channel height	*H*	1 or 1.2	mm
Gas bubble length	*L*	2H to 6H	
Excitation frequency	*f*	500	kHz

### 2.2 Classification of phenomena

**Fig. 1:**
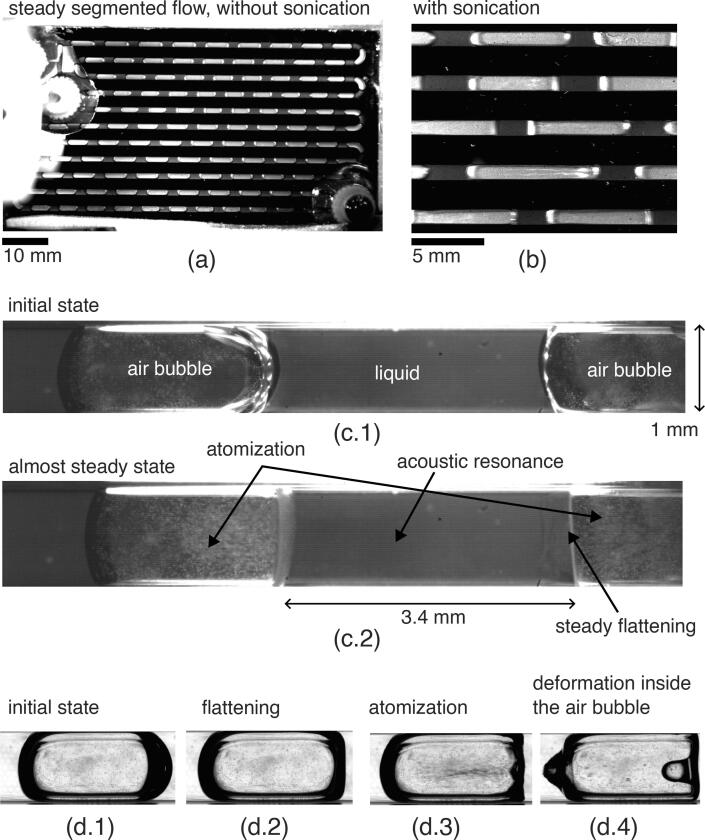
Illustration of phenomena generated by acoustic resonance between gas bubbles in a microreactor. (a): Overview of the microreactor with segmented flow, without sonication. (b): overview of the effect of ultrasound on gas bubbles. (c.1) and (c.2): Steady flattening with atomization. (d.1) to (d.4): Transient deformation with atomization. For these examples, the excitation frequency was around 440 kHz.

From the experimental observations in Mc Carogher et al. [27], it is possible to classify the different dynamic behaviors driven by acoustic resonance as follows (see also Fig. 1 for illustration):

• Steady flattening of the gas bubble
• Strong transient shock on the gas bubble
• Nonlinear oscillations of the gas bubble
• Atomization at the sonicated surface of the gas bubble

In general, interface flattening occurs prior to atomization. As illustrated in Figs. 1 (c.1) and (c.2), flattening can be observed with or without atomization at the flattened interface. All of these observations highlight resonance order in the channel axis. The cutoff frequency for the existence of high order resonance in the channel cross section is $f_{c}=c_{0}/(2h)=$ 617 kHz, for a 1.2 × 1.2 mm channel, and 740 kHz for a 1.0 × 1.0 mm channel. The selected frequency was thus below this first cutoff frequency at which a pressure node would appear at the mid-channel height.

**Fig. A.26:**
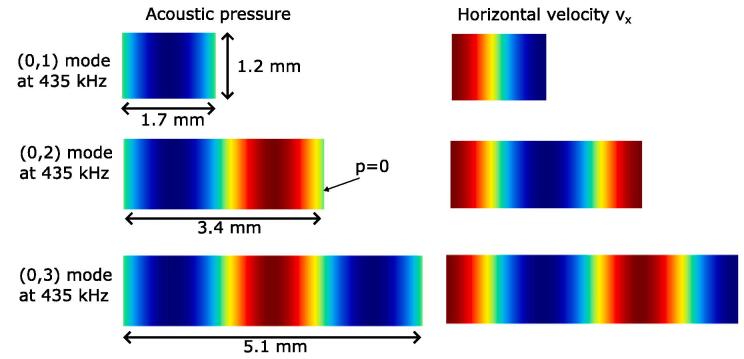
Resonant liquid lengths for a 435 kHz excitation frequency. The red-green-blue color scale is used, where green stands for zero.

**Fig. A.27:**
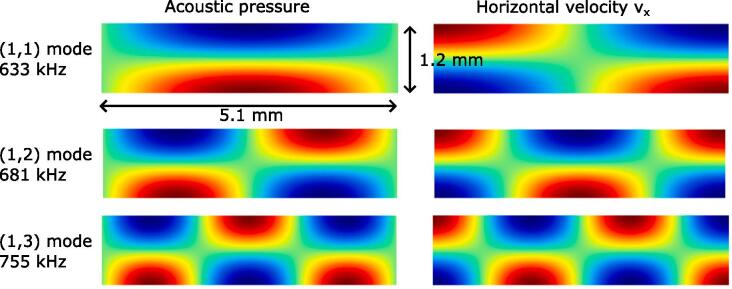
Possible higher order modes for a fixed liquid length. The red-green–blue color scale is used, where green stands for zero.

It is possible to define a mode using the notation (n,m), where n∈{1,2,…} stands for the order along the channel axis and m∈{0,1,2,…} stands for the order along the two axes of the square cross section. Since the gas–liquid interface may be approximated as a free surface, with a zero acoustic pressure boundary condition, the liquid acts as an open acoustic resonator. Examples of mode shapes of interest are given in Appendix A in Fig. A.26, Fig. A.27.

### 2.3 Nonlinear acoustics modeling

In linear acoustics, the average motion of a particle oscillating in an acoustic field is null. The non-zero average motion is a second-order nonlinear effect and is described as the effect of the acoustic radiation force^1^. According to Baudoin and Thomas in their review on particle and fluid micromanipulation, a definition of the acoustic radiation force would be: a net force applied at the interface between two media with different acoustic properties, this force enables manipulations of particles but also deformation of fluid interfaces [28]. Since a standing acoustic wave is determined by boundaries, which act as reflectors, moving boundaries result in a dynamic acoustic field. As a consequence, the considered configuration involves an additional type of nonlinearity, which is the deformation of the domain where the acoustic field is set. The acoustic radiation force is directly determined by the acoustic field via its amplitude and pattern (nodes and antinodes location, focusing). A large acoustic amplitude occurs when resonance conditions are met. Consequently, when the system changes over time, resonance conditions vary and can be met at specific instants. This can be summarized under the concept of *dynamic acoustic resonance*.

Dynamic acoustic resonance effects have been addressed by Simon et al. for the acoustic fountain configuration [29], [30]. Other authors have studied similar configurations [31]. The focusing of a standing ultrasonic field, generated by a focused ultrasound transducer, on a free liquid surface induces a rise of the liquid. When the height equals a specific fraction of the wavelength, a sudden local resonance produces droplet formation. The sequence of resonances can give rise to a droplet chain growing vertically. We noticed that the principle involved in Simon’s configuration was similar to the one encountered in our microreactor. Contrary to Simon’s configuration, in the present study, the high acoustic amplitude is not obtained by focusing but by pure resonance. In such a configuration, the acoustic radiation force effects are much more significant than acoustic streaming effects.

### 2.4 Derivation of the acoustic radiation force acting on a free surface

The explicit formula for the acoustic radiation force acting on a gas bubble boundary can be derived from the perturbation expansion method. This method is commonly used for acoustic streaming and particle manipulation prediction, see *e.g.* [32], [33], [34], [35], [36]. Let us consider a liquid domain Ω, whose boundary is composed of walls $\Gamma_{\mathrm{walls}}$, and free surface $\Gamma_{\mathrm{free}}$. Writing the expansions of pressure, velocity and density, $p=p_{0}+p_{1}+p_{2},v=v_{0}+v_{1}+v_{2},\rho =\rho_{0}+\rho_{1}+\rho_{2}$, respectively, the first order acoustic equations defined in Ω are

(1) $$\frac{1}{c_{0}^{2}}\partial_{t}p_{1}+\rho_{0}\nabla \cdot v_{1}=0,$$

(2) $$\rho_{0}\partial_{t}v_{1}=-\nabla p_{1},$$

(3) $$\rho_{1}=\frac{1}{c_{0}^{2}}p_{1},$$

for $v_{0}=0$ (fluid at rest relative to the reference frame) and $p_{0}=0$ (relative to the static pressure, comprising equilibrium surface tension pressure and fluid pressure). The speed of sound in the liquid is denoted by $c_{0}$. The boundary layer is neglected, and a slip condition is set at $\Gamma_{\mathrm{walls}}$. Since one can show that the capillary wave and acoustic wave solutions are uncoupled, assuming a pure acoustic wave in Ω, the boundary condition at $\Gamma_{\mathrm{free}}$ can be approximated simply by p=0.

The time-averaged second order equation assuming an incompressible and inviscid fluid and the pure harmonic solution of the first order equations defined in Ω are

(4) $$\nabla \cdot v_{2}=0,$$

(5) $$\rho_{0}\partial_{t}v_{2}=-\nabla p_{2}-\rho_{0}\langle \nabla \cdot v_{1}⊗v_{1}\rangle_{T},$$

where $\langle \cdot \rangle_{T}$ denotes time averaging over a period *T*. Since a change of the free boundary geometry from the equilibrium geometry is expected, a resulting capillary force emerges. The boundary condition at $\Gamma_{\mathrm{free}}$ for the second-order system is $p=-\gamma \Delta_{s}\eta_{2}$, where $\eta_{2}$ represents the normal displacement at the surface (pointing outwards of Ω), γ is the surface tension, and $\Delta_{s}$ denotes the surface Laplacian. Finally, assuming no additional forces other than surface tension, one obtains the following synthetic equation at $\Gamma_{\mathrm{free}}$:

(6) $$\underset{\mathrm{inertia}}{\underset{︸}{\rho_{0}\partial_{t}v_{2}}}=\underset{\mathrm{capillary force}}{\underset{︸}{\gamma \nabla \Delta_{s}\eta_{2}}}\underset{\mathrm{acoustic force}}{\underset{︸}{-\rho_{0}\langle \nabla \cdot v_{1}⊗v_{1}\rangle_{T}}}\text{.}$$

The acoustic radiation force acting on a free surface can be also derived from Zakharov and Filonenko’s modeling of capillary wave turbulence at the surface of a semi-infinite fluid [37], [38]. The modeling involves the normal displacement of the surface η, the scalar velocity potential (irrotational) ψ, defined at the surface and in the fluid. Considering a two-dimensional domain (thus a one-dimensional surface) and applying the perturbation method the linear capillary wave model is:

(7) ∂tη1=∂^xψ1,

(8) $$\rho_{0}\partial_{t}\psi_{1}=\gamma \partial_{\mathrm{xx}}\eta_{1},$$

where *x* stands for the space variable along the one-dimensional surface. ∂^x denotes the one-dimensional 1/2 fractional Laplacian which can be defined, for an infinite surface, by the Fourier transform:

(9) ∂^xf=12π∫-∞∞|k|F(k)eikxdk,

with *F* being the Fourier transform^2^ of the function *f*. Eqs. (7), (8) describe linear capillary waves whose dispersion relation is $\rho_{0}\omega^{2}=\gamma k^{3}$. Applying the expansion method while assuming a purely harmonic solution of the first order system, the second order time averaged equations of Zakharov and Filonenko’s model are:

(10) ∂tη2=∂^xψ2,

(11) ρ0∂tψ2=γ∂xxη2-ρ012〈∂xψ12-∂^xψ12〉T,

Eqs. (10), (11) can be combined to obtain the following synthetic equation:

(12) ρ0∂ttη2︸inertia=γ∂^x∂xxη2︸capillary force-ρ012∂^x〈∂xψ12-∂^xψ12〉T︸acoustic force,

Eqs. (6), (12) provide two different frameworks to desribe the acoustic deformation of surfaces, which consist of a balance between inertia, surface tension and the acoustic radiation force. Both modeling approaches give the same result for the case of the acoustic deformation of an infinitely long liquid film of a given initial depth, as shown in Appendix B. This case has been studied by Scortesse et al. [39], where a similar expression for the acoustic radiation force in a liquid film was derived.

### 2.5 Numerical scheme involving an updated standing acoustic field

**Fig. 2:**
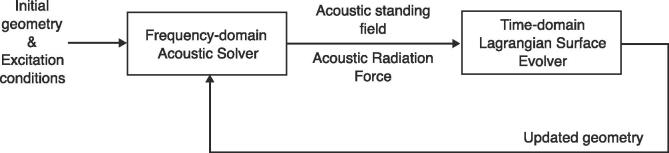
Structure of the selected numerical method.

The selected numerical scheme for dealing with dynamic acoustic resonance in two-phase media is summarized in Fig. 2. The main challenges of the definition of the numerical method are: (i) a relatively strong nonlinear behaviour is expected, (ii) dealing with a large acoustic amplitude range, (iii) update of the computational domain (*i.e.* remeshing). The main idea of our approach was to combine a frequency-domain acoustic solver with a time-domain surface Lagrangian solver for the deformation of the gas bubble. This approach is valid if the establishment time of the standing acoustic wave is much smaller than the characteristic deformation time of the surface. Since the typical maximum velocity of the observed moving interfaces is in the range of 2 m/s, with the speed of sound in water being around 1480 m/s, one can propose that this is a reasonable approximation. The solvers are described in detail in the following paragraphs.

### 2.6 The 2D approximation

**Fig. 3:**
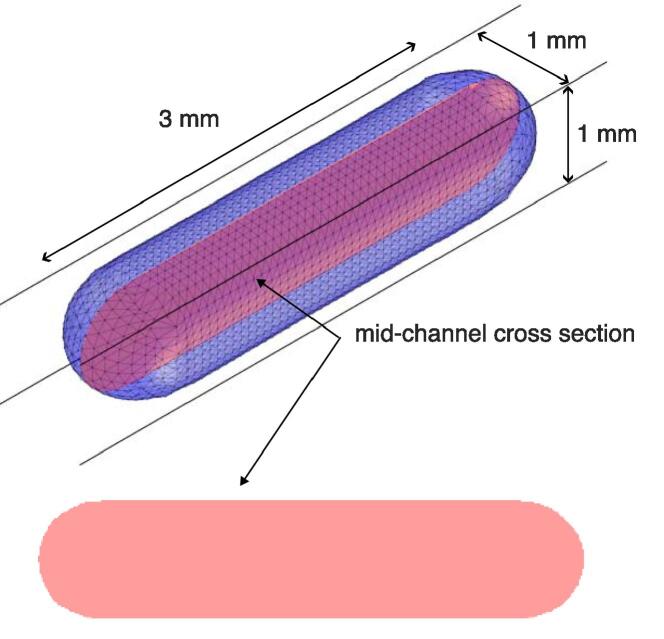
2D approximation of a 3D gas bubble in a square cross section channel.

A two-dimensional approximation was chosen to model the gas bubble, which represents a slice of the actual three-dimensional gas bubble, as illustrated in Fig. 3. The gas bubble was thus modeled as a closed curve in the x,y plane. This choice of a 2D model has two motivations. Firstly, the relative simplicity of the acoustic field does not imply significant 3D motion, in the sense that the motion can be reduced in the channel axis and in one of the cross section plane dimensions of the channel due to symmetry. Secondly, the 2D model allowed to perform a large set of simulations with fine mesh both in time and space, which aided the complete analysis.

### 2.7 The frequency-domain acoustic solver

**Fig. 4:**
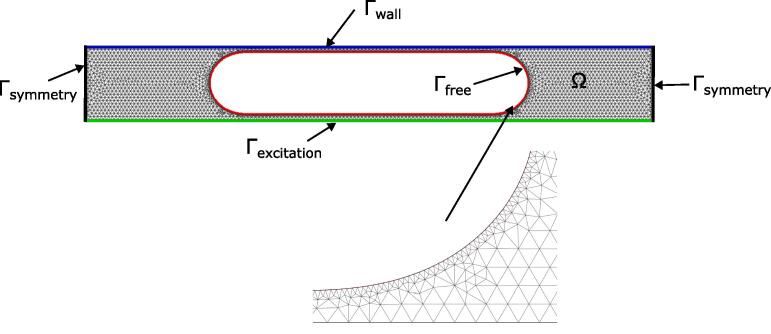
Typical configuration for the two-dimensional finite element acoustic solver. In this example, excitation is set at the bottom.

The linear acoustic equations are reduced to the frequency-domain pressure formulation:

(13) $$\Delta p+\frac{\omega^{2}}{c_{0}^{2}}p=0,\;\mathrm{in}\;\Omega ,$$

where ω is the pulsation of the monochromatic acoustic wave (in rad/s). The boundary conditions are shown in Fig. 4, which were: p=0 at $\Gamma_{\mathrm{free}},\nabla p\cdot n=0$ at $\Gamma_{\mathrm{fwall}}$ and $\Gamma_{\mathrm{symmetry}}$, and $\nabla p\cdot n=\rho_{0}\omega^{2}u_{a}$ at $\Gamma_{\mathrm{excitation}}$, where $u_{a}$ is the oscillation amplitude of the given excitation. Due to the significant acoustic mismatch between air and water, the acoustic field transmitted inside the gas bubble can be neglected.

A common finite element method (FEM) was implemented. For a given gas bubble geometry the domain Ω was meshed with triangular elements. The size of the regular mesh elements was 0.6e-4 m, and the size of the fine mesh elements at the bubble interface was 1.5e-5 m (the mesh is illustrated in Fig. 4). Linear elements with three integration points where used. The computed acoustic field is then assumed to be in the form $p_{1}(x,y,t)=p(x,y)\sin(\omega t)$, where p(x,y) is the solution of the frequency domain problem. Time averaging, when computing the acoustic radiation force, introduces a 1/2 factor from $\langle \sin^{2}(\omega t)\rangle_{T}$ or $\langle \cos^{2}(\omega t)\rangle_{T}$. The particular oscillation velocity $v_{1}$ and amplitude $u_{1}$ were computed according to:

(14) $$\rho_{0}i\omega v_{1}=-\nabla p_{1},$$

(15) $$i\omega u_{1}=v_{1}\text{.}$$

The resonance consists of a singularity in the sense that the acoustic field is not defined at the resonance frequency. In practice, the acoustic field at resonance has a finite value because of physical saturation. To tackle the singularity, a loss factor of 5e-4, set empirically, was chosen for artificial dampening in the following results.

### 2.8 Lagrangian surface evolver approach

A Lagrangian “Surface Evolver” was implemented in MATLAB (version R2021a) to capture the gas bubble deformation, which is a similar method as the Surface Evolver by Kenneth Brakke [40], [41]. The method consists of considering the motion of the surface driven by surface tension, external forces and constraints, without computing the flow field in the surrounding fluids. The prediction of the motion of the liquid in the bulk domain was not of essential interest compared to the motion of the interface. One of the benefits of such an approach is that it makes the computation straightforward, since the number of nodes is considerably reduced, which allowed us to perform a large number of simulations.

The closed curve associated with the gas bubble geometry was meshed by evenly spaced nodes. The cell size was 1.5e-5 m. Volume conservation was ensured by adding a constraint to the numerical area enclosed by the curve at each time step, which then also approximates the incompressibility condition. As a consequence of the Surface Evolver approach, Zakharov’s formulation in Eq. (12) was used, which is valid for closed surfaces. The fractional Laplacian in Eq. (9) is then transposed to its periodic domain equivalent involving a Fourier series form. A central difference scheme and an explicit strategy was used for the acoustic and capillary forces, so that the latter are computed according to the current time step only. For each node position x=(x(t),y(t)) of the surface the Lagrangian integration had the following form:

(16) $$x_{t+1}=2x_{t}-x_{t-1}+\frac{\Delta t^{2}}{\rho_{0}}\left(f_{\mathrm{capillary},t}+f_{\mathrm{acoustic},t}\right)n_{t},$$

where the subscripts denote the time step indexes, Δt the time step, and $n_{t}$ represents the outward liquid surface normals at the current time step. The capillary force was computed by

(17) fcapillary,t=γ∂^sκt.

The curvature at the current time step $\kappa_{t}$ is given explicitly by

(18) $$\kappa_{t}=\frac{\left(\frac{{\mathrm{dx}}_{t}}{\mathrm{ds}}\frac{d^{2}y_{t}}{{\mathrm{ds}}^{2}}-\frac{{\mathrm{dy}}_{t}}{\mathrm{ds}}\frac{d^{2}x_{t}}{{\mathrm{ds}}^{2}}\right)}{{\left({\left(\frac{{\mathrm{dx}}_{t}}{\mathrm{ds}}\right)}^{2}+{\left(\frac{{\mathrm{dy}}_{t}}{\mathrm{ds}}\right)}^{2}\right)}^{\frac{3}{2}}},$$

where *s* denotes the local coordinate of the curve. The computation of the fractional Laplacian in Eq. (17) was done using the Fast Fourier Transform (fft) equivalent of Eq. (9). For a given current solution of the frequency-domain acoustic solver, the acoustic force $f_{\mathrm{acoustic},t}$ was computed according to the third term in Eq. (12).

The channel walls were modeled as a force preventing penetration of the surface through them. The setting of the force constant of the walls was done empirically according to the equilibrium shape problem of a gas bubble in a channel (one result of the equilibrium shape problem is provided in the Supplementary Material, see reference in Appendix D).

When dealing with strong deformation mesh distortion arises, hence a remeshing strategy is necessary to ensure mesh regularity. The main issue with remeshing of a surface during motion is the error and possible aberrations coming from the interpolation of the speed from the current mesh to the next one. This error can be reduced by reducing the mesh size. We noticed that the interpolation error is quite dependent on the remeshing partitioning strategy. One way to define partitioning is to define Lagrangian trackers that are not affected by the remeshing and to define the partition of the surface according to them. While computing the motion, one needs to update the acoustic force according to the new boundary. In this study, updating time steps from 5e-6 s (fine) to 1e-4 s (coarse) were chosen.

To validate the selected solver, comparisons with OpenFOAM volume of fluid (VOF) simulations were performed for the following initial shape problems: (i) merging of a droplet on a flat surface, (ii) bump on a flat surface. Another comparison was also done for the case of the rising droplet based on Hua and Lou’s benchmark [42]. Videos of these preliminary simulations are provided in the Supplementary Material, see Appendix D. In general, the behavior of the interface is well reproduced and the surface solver could deal with strongly concave shapes in a stable way.

### 2.9 Methodology for atomization prediction

Atomization consists of the breakup of bulk liquid in multiple ejected droplets at a free surface. As observed by Mc Carogher et al. [27], different mechanisms of atomization are involved in a gas–liquid microreactor. These can be divided into three types: (*i*) capillary, (*ii*) cavitation, and (*iii*) coupled capillary-cavitation. The focus of this work is on the Faraday instability, which belongs to the capillary type, as one of the mechanisms resulting in atomization. In the two-phase microreactor, the (0,n)-mode standing wave results in an oscillation of the bubble interface in the channel axis direction at the ultrasonic excitation frequency. This oscillation, when reaching a large amplitude, leads to the Faraday instability and, at some point, to atomization. Based on Puthenveettil and Hopfinger’s analysis of the oscillating tank experiment [43], one can divide the capillary atomization generation mechanism into 4 stages associated with different excitation amplitude ranges:

• Linear regime
• Standing subharmonic nonlinear Faraday wave
• Chaotic motion below the atomization threshold
• Atomization

**Fig. 5:**
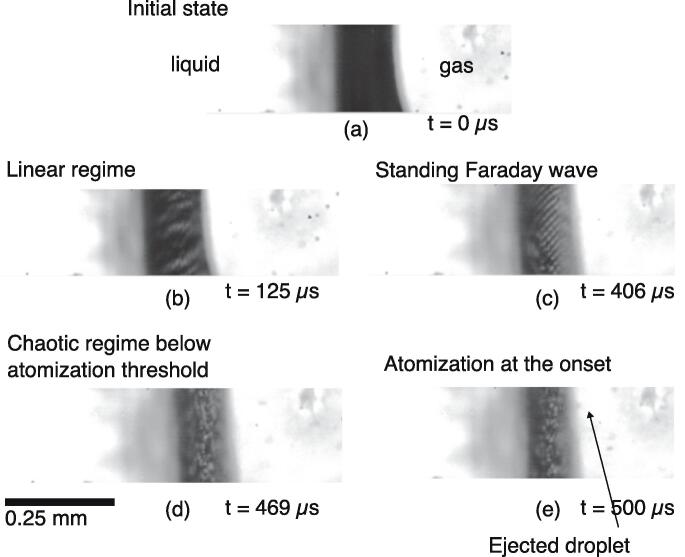
Illustration of atomization generation stages at a gas bubble interface exposed to a resonant standing wave in a microreactor. Artificial numerical contrast was applied to the images. The images are taken from a 32,000 fps video highlighting a small part of the interface, see Video 1 in the ESI.

The same stages are observed in the ultrasonic microreactor, as illustrated in Fig. 5 and in Video 1 (ESI).

**Fig. 6:**
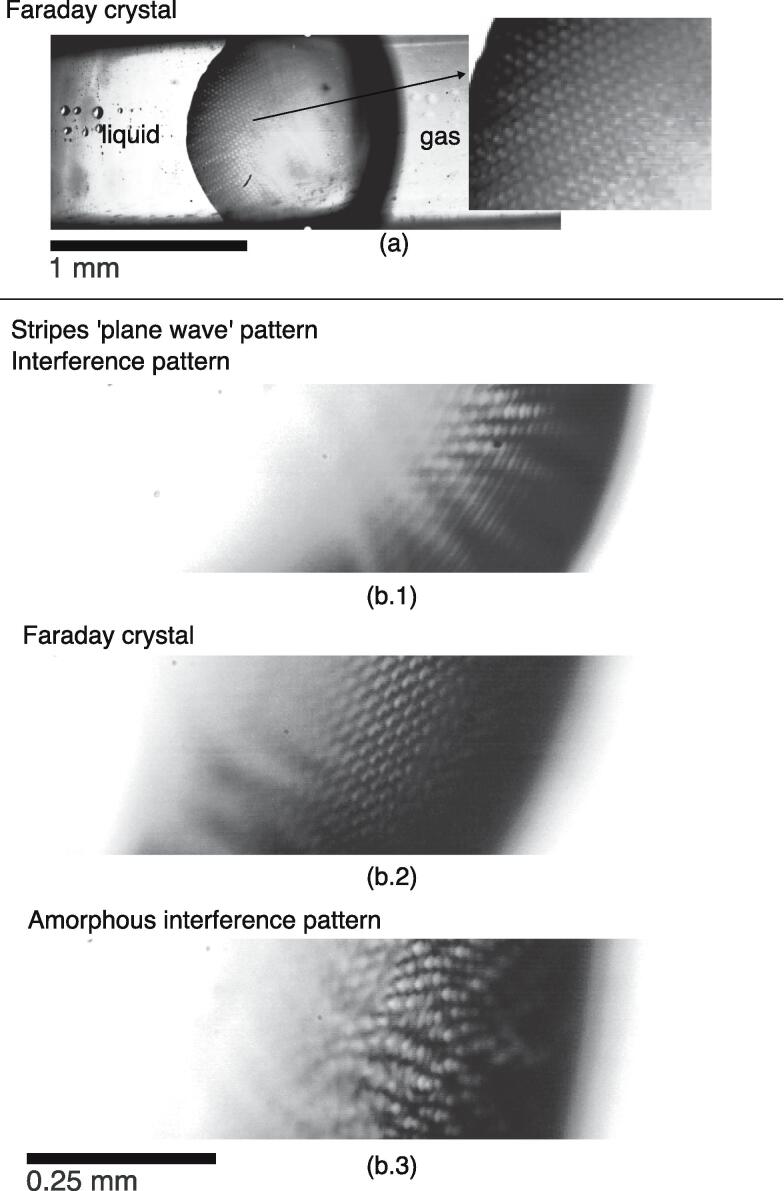
Different standing wave patterns observed at a sonicated gas bubble interface (taken from Videos 2 and 3 in the ESI).

In the linear regime, a standing linear capillary wave oscillating at the driving frequency can be excited at the surface. In this regime, the superposition of waves is valid and simple patterns, such as a plane wave or interference patterns, can be observed. Depending on the configuration, the linear capillary wave can be caused by the presence of a contact point with a vibrating solid surface, as in the vibrating tank experiment. Above a threshold, a standing subharmonic Faraday wave is observed. Faraday crystals can form at the gas bubble surface, where mostly the square pattern is observed, as shown in Fig. 5 (c). The chaotic stage is characterized by an unstructured surface deformation with salient crests. Finally, atomization happens when breakup occurs at the crests, and at the onset of breakup ligament or elongated droplets can be observed. Fig. 6 provides an overview of the different standing wave patterns observed in the experiments.

**Fig. 7:**
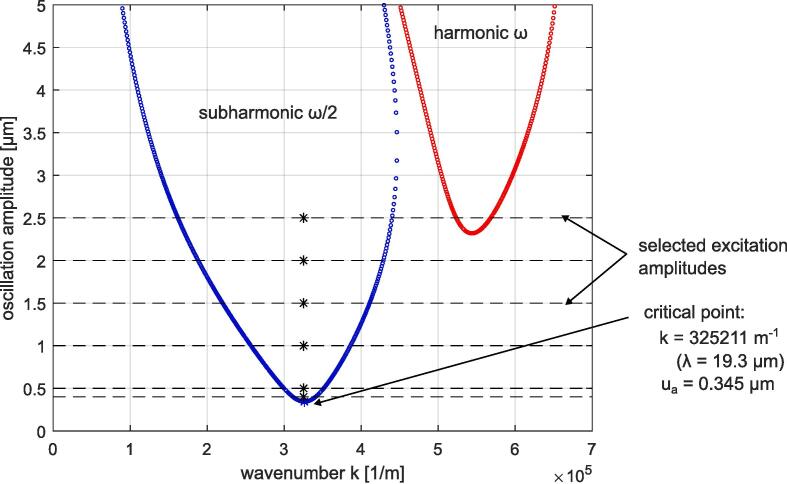
Kumar and Tuckerman instability tongues for an air–water interface oscillating at 500 kHz.

The theoretical description of the Faraday instability was developed by Kumar and Tukerman [44]. The Kumar and Tuckerman theory (K&T theory) is based on analytical solutions of the Navier–Stokes equations for the interface between two fluids (of finite or infinite depth) oscillating at a given driving frequency. The K&T theory is known to be an accurate method to predict the instability threshold. It is possible to define the Faraday instability threshold with respect to the critical point of the first subharmonic instability region. This corresponds physically to the emergence of a standing wave oscillating at ω/2, where ω is the driving pulsation (in rad/s). The critical point is determined by the dynamic viscosity of the two fluids and their interfacial tension. In case of an air–water interface the effect of the air viscosity is negligible. The instability chart for an air–water interface with a driving oscillation frequency of 500 kHz is depicted in Fig. 7. The oscillation is assumed to be a pure harmonic forced displacement of the interface, of the form $u=u_{a}\sin(\omega t)$, so that the magnitude of velocity and acceleration are simply $\omega u_{a}$ and $\omega^{2}u_{a}$, respectively. The critical wavenumber is 325211 m^−1^, resulting in a critical wavelength of 19.3 μm (k=2π/λ). The critical oscillation amplitude is 0.345 μm. The selection of the oscillation amplitudes for the numerical analysis was based on this theoretical value.

**Fig. 8:**
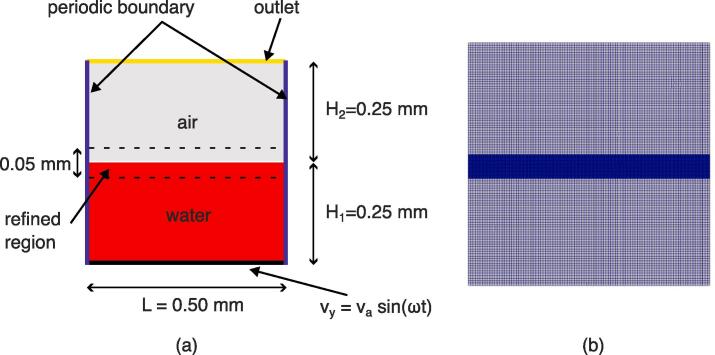
(a): Schematic of the selected VOF simulation. (b): View of the selected mesh.

Two-dimensional volume of fluid (VOF) simulations were performed to aid the atomization prediction. For this, the standard OpenFOAM solver *interFoam* was modified to include a smoothing method to suppress spurious currents (see [45] for solver details). The geometry consisted of a rectangular 2D tank with a driven vibrating bottom (oscillating at 500 kHz for different oscillation amplitudes, which were: 0.4 μm, 0.5 μm, 1.0 μm, 1.5 μm, 2.0 μm, and 2.5 μm) and periodic boundary conditions at the sides. To enable the vibration of the bottom wall, a dynamic mesh was applied using the *displacementLaplacian* solver, which solves cell motion based on the Laplacian of the diffusivity and the cell displacement, see [46] for details. The schematic of the configuration with dimensions is shown in Fig. 8 (a), and the fluid properties are given in Table 1. This 2D approximation allowed to perform the different simulations associated with different excitation amplitudes within a reasonable computation time. The mesh was generated using *blockMesh* and then *refineMesh* was implemented to refine the mesh near the interface (for a thickness of 5e-5 m), see Fig. 8 (b). The final mesh consisted of 50360 cells with a cell size of 4.7e-6 m in the regular region, and 1.3e-6 m in the refined region. The time step size was automatically changed based on a maximum Courant number of 0.2. These VOF simulations enabled the prediction of the air–water interface behavior oscillating at 500 kHz for the different oscillation amplitudes specified above, and their position against theoretical instability tongues are indicated in Fig. 7.

## 3 Results

### 3.1 Natural deformation patterns

**Fig. 9:**
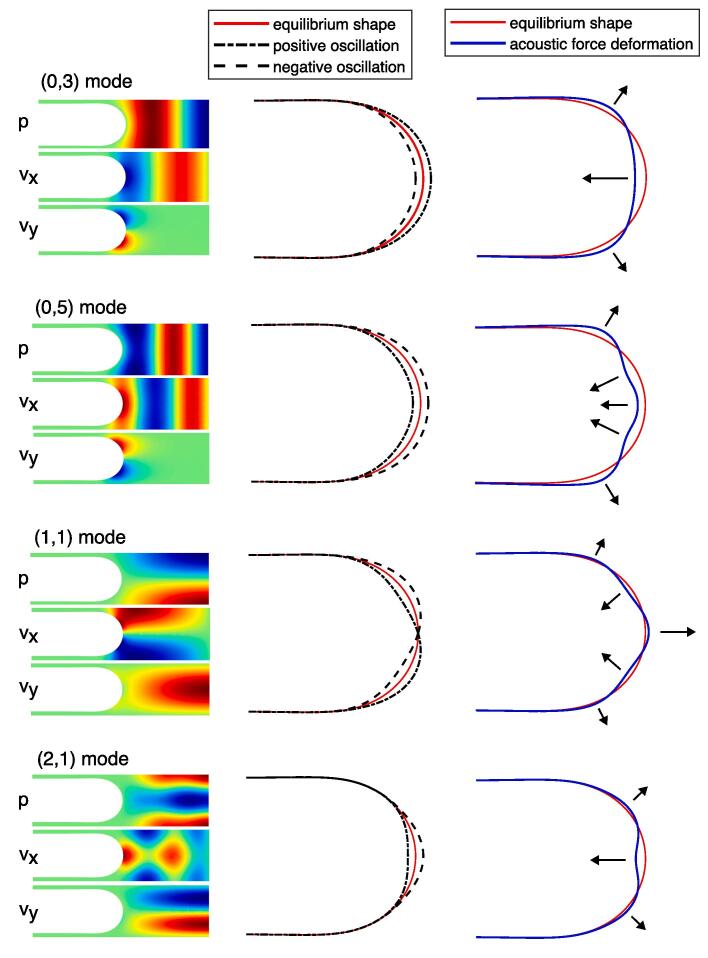
Qualitative view of natural deformation patterns for associated acoustic modes between gas bubbles. The mode index is indicated. First column: qualitative view of the acoustic field with pressure *p*, horizontal velocity $v_{x}$ and vertical velocity $v_{y}$. Second column: oscillation of the surface. Third column: deformation pattern due to the acoustic radiation force. The red-green-blue color scale is used, where green indicates zero.

First, a qualitative view of the natural deformation patterns associated with particular modes is given in Fig. 9, which highlights the dependence of the deformation of the sonicated interface on the resonance mode. The (0,1) and (0,2) modes (not displayed) have a similar deformation pattern as the (0,3) mode. This particular deformation pattern consists of a flattening of the interface. The middle of the interface is pushed towards the inside of the gas bubble while the sides are pulled towards the walls. For the (1,1) mode, the middle of the bubble is pulled towards the liquid, leading to a bump instead of flattening. The number of bumps–concavities increases with the order of the mode in the channel cross section. A bump towards the liquid is associated with a pressure node near the interface, while a concavity is associated with a pressure antinode near the interface.

### 3.2 One-side transient flattening on free bubble

**Fig. 10:**
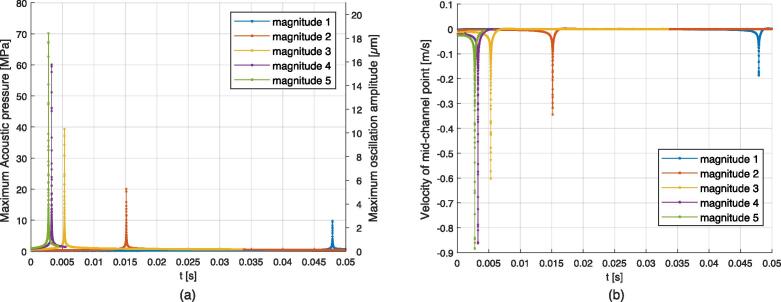
Simulations of one-side transient flattening on a free bubble. (a): Evolution of the magnitude of the acoustic field expressed as maximum pressure and particular oscillation amplitude. (b): Evolution of the velocity at the mid-channel point of the gas bubble.

**Fig. 11:**
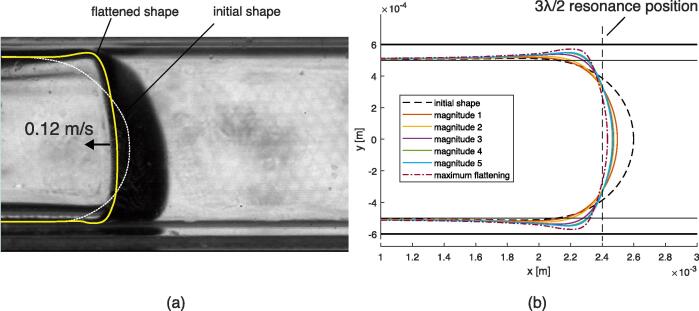
Qualitative comparison of the gas bubble flattening profile. (a): Observation taken from Video 4 (frame No. 75). (b): Simulation results for one-side resonance for different magnitudes. The 3λ/2 resonance position corresponds to the distance to the right border (no displayed) matching the length of ideal rectangle resonator (0,3) mode.

First, the simulation results for the case of a gas bubble without flow constraint submitted to a 500 kHz one-side resonance are discussed. The excitation was located on the right boundary to excite the (0,3) mode, and the simulation parameters for 5 different excitation magnitudes are provided in C in Table C.6. Video 8 illustrates the obtained behavior for the magnitude 4 case, and additional videos in the Supplementary Material show the evolution of gas bubble and acoustic field magnitude over time, see reference in Appendix D. The evolution of the acoustic magnitude, given in terms of maximum acoustic pressure and maximum particular oscillation amplitude, and the mid-channel point (y=0) velocity, are depicted in Fig. 10. For each magnitude, a resonance peak emerges resulting in interface flattening near the ideal rectangle resonator length for (0,3) mode, which in this case is at about x=2.4 mm. The velocity of the mid-channel point quantifies the speed of this deformation, and the obtained values for each simulation are provided in Table 2. The higher magnitudes being pushed faster to the resonance position, it can be seen that the associated resonance peak is reached sooner than for lower magnitudes. The flattening shapes are compared to experimental results corresponding to Video 4 in Fig. 11. The measured flattening velocity for this experiment was about 0.12 m/s. The flattening magnitude increases with the acoustic field magnitude, from weak deformation to a strongly flattened shape. An acoustic pressure peak in the range of 10 MPa is needed to produce a visible transient flattening, which requires a velocity in the range of 0.2 m/s. A maximum flattening magnitude is obtained for this configuration, see associated shape in Fig. 11 (b). This limit is reached for a large acoustic magnitude exceeding 80 MPa. For these elevated pressure values a high dynamic range is obtained in a short time of about 1e-5 s. After the flattening, the gas bubble, in this particular free condition, moves away from the resonance position due to inertia.

**Table 2:** Output values measured for the one-side transient flattening on a free gas bubble simulations. $|v_{2}{|}_{\max}$: maximum deformation velocity. $|p_{1}{|}_{\max}$: maximum acoustic pressure. $|u_{1}{|}_{\max}$: maximum acoustic particular oscillation amplitude. width $p_{1}$: width of the acoustic resonance peak determined at $0.5\cdot |p_{1}{|}_{\max}$.

Configuration Ref.	$|v_{2}{|}_{\max}$	$|p_{1}{|}_{\max}$	$|u_{1}{|}_{\max}$	width $\left|p_{1}\right|$
Magnitude 1	0.188 m/s	9.80 MPa	2.81 μm	3.9e−5 s
Magnitude 2	0.344 m/s	20.07 MPa	5.63 μm	2.0e−5 s
Magnitude 3	0.602 m/s	39.35 MPa	10.72 μm	1.15e−5 s
Magnitude 4	0.863 m/s	60.11 MPa	16.18 μm	0.7e−5 s
Magnitude 5	0.885 m/s	70.18 MPa	18.82 μm	1.0e−5 s

### 3.3 One-side transient flattening on constrained bubble

**Fig. 12:**
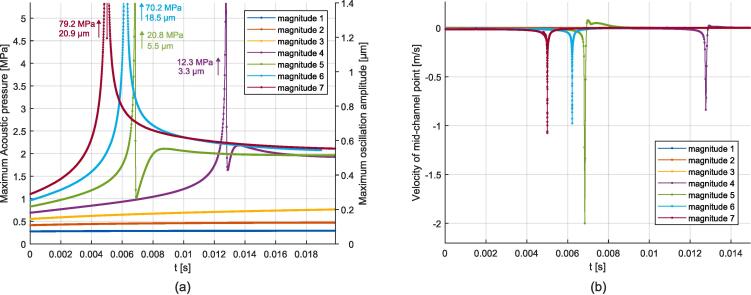
Simulations of one-side transient flattening on a constrained gas bubble. (a): Evolution of the magnitude of the acoustic field expressed in maximum pressure and particular oscillation amplitude (magnitude 3 peak is not visible). (b): Evolution of the velocity at the mid-channel point of the gas bubble.

To take into account the presence of the surrounding liquid in the channel, a volume constraint on both sides of the gas bubble was added to the previous configuration. Accordingly, the gas bubble is unable to move away from the resonance position. Seven excitation magnitudes were chosen, see Table C.7. Video 9 illustrates the obtained behavior for the magnitude 4 case, and additional videos in the Supplementary Material show the evolution of the gas bubble and acoustic field magnitude over time, see reference in Appendix D. The plots of the acoustic magnitude are shown in Fig. 12. The introduction of the volume constraint leads to a different behavior compared to the free bubble case. After a resonance peak, a steady state is reached. The acoustic field stabilizes at around $\left|p_{1}\right|$ = 2 MPa, $\left|u_{1}\right|$ =  0.55 μm for magnitudes 3 to 7, see Table 3. For the selected simulation time, no peak is observed for magnitudes 1 and 2, as the transient deformation towards the resonance position is too low to reach a peak.

**Table 3:** Output values measured for the one-side transient flattening on a constrained gas bubble simulations. $|v_{2}{|}_{\max}$: maximum deformation velocity. $|p_{1}{|}_{\max}$: maximum acoustic pressure. $|u_{1}{|}_{\max}$: maximum acoustic particular oscillation amplitude. $\left|p_{1}\right|$ end: end value of acoustic pressure. $\left|u_{1}\right|$ end: end value of particular acoustic oscillation amplitude.

Configuration Ref.	$|v_{2}{|}_{\max}$	$|p_{1}{|}_{\max}$	$|u_{1}{|}_{\max}$	$\left|p_{1}\right|$ end	$\left|u_{1}\right|$ end	
Magnitude 1	0.0012 m/s	0.29 MPa	0.09 μm	0.29 MPa	0.09 μm	
Magnitude 2	0.0027 m/s	0.47 MPa	0.14 μm	0.47 MPa	0.14 μm	
Magnitude 3	0.5053 m/s	7.96 MPa	2.11 μm	1.85 MPa	0.48 μm	
Magnitude 4	0.8379 m/s	12.26 MPa	3.25 μm	1.90 MPa	0.49 μm	
Magnitude 5	2.0001 m/s	20.84 MPa	5.51 μm	1.96 MPa	0.51 μm	
Magnitude 6	0.9772 m/s	70.19 MPa	18.49 μm	2.07 MPa	0.53 μm	
Magnitude 7	1.0750 m/s	79.21 MPa	20.86 μm	2.07 MPa	0.53 μm	

### 3.4 Both-sides resonance

**Fig. 13:**
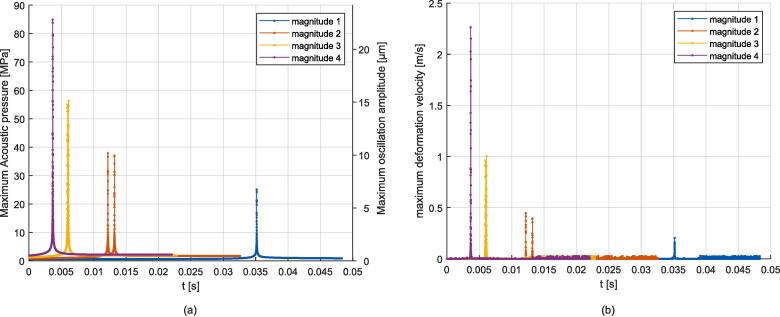
Simulations of both-sides resonance. (a): Evolution of the magnitude of the acoustic field expressed in maximum pressure and particular oscillation amplitude (magnitude 3 peak is not visible). (b): Evolution of the maximum velocity of the gas bubble deformation.

**Fig. 14:**
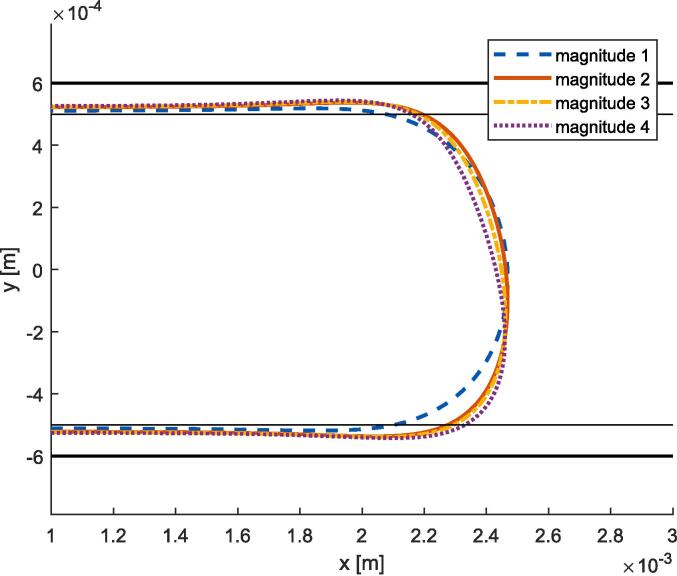
Comparison of steady flattening of gas bubble deformation for the both sides resonance obtained with bottom excitation.

In this section, simulation results for the case of both-sides resonance are presented. Unlike the previous 2 configurations, the excitation is now applied at the bottom boundary, for which four excitation magnitudes were selected, see Table C.8. Consequently, this configuration does not exhibit symmetry along the mid-channel axis anymore, and acoustic standing waves appear on both sides of the centered gas bubble. Video 10 illustrates the obtained behavior for the magnitude 3 case, and additional videos are provided in the Supplementary Material, see reference in Appendix D. As the initial mesh is not perfectly symmetric, a delay is observed between the left and right resonance peaks, see for example the magnitude 2 curve in Fig. 13. After the two resonance peaks, a steady state is reached. The comparison of steady flattening of the right side for the different magnitudes is shown in Fig. 14, and the acoustic field and deformation values are summarized in Table 4.

**Table 4:** Output values measured for the both-sides resonance on a gas bubble simulations. $|v_{2}{|}_{\max}$: maximum deformation velocity. $|p_{1}{|}_{\max}$: maximum acoustic pressure. $|u_{1}{|}_{\max}$: maximum acoustic particular oscillation amplitude. $\left|p_{1}\right|$ end: end value (steady) of acoustic pressure. $\left|u_{1}\right|$ end: end value (steady) of particular acoustic oscillation amplitude.

Configuration Ref.	$|v_{2}{|}_{\max}$	$|p_{1}{|}_{\max}$	$|u_{1}{|}_{\max}$	$\left|p_{1}\right|$ end	$\left|u_{1}\right|$ end
Magnitude 1	0.20 m/s	25.0 MPa	6.9 μm	0.6 MPa	0.13 μm
Magnitude 2	0.45 m/s	37.9 MPa	10.1 μm	1.7 MPa	0.38 μm
Magnitude 3	1.00 m/s	56.3 MPa	15.0 μm	1.9 MPa	0.40 μm
Magnitude 4	2.26 m/s	84.9 MPa	22.5 μm	2.2 MPa	0.43 μm

### 3.5 One-side resonance on an advected gas bubble

**Fig. 15:**
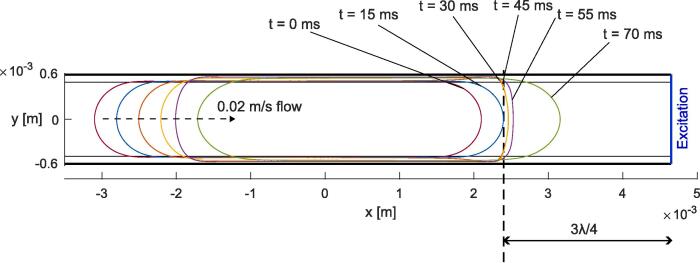
Evolution of the gas bubble shape for the case of the advected gas bubble exposed to one-side resonance for the case of magnitude 4.

**Fig. 16:**
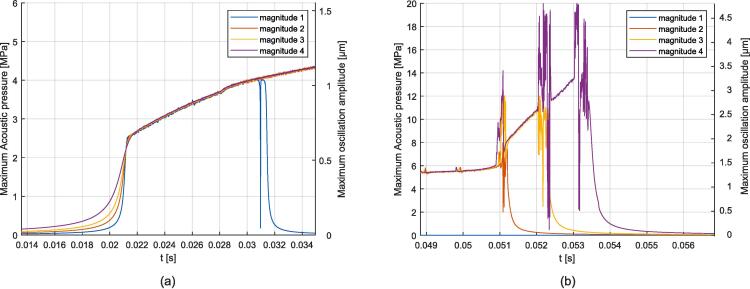
Evolution of the acoustic amplitude over time for the case of the advected gas bubble exposed to one-side resonance. (a) Start of the acoustic resonance. (b) End of acoustic resonance.

**Fig. 17:**
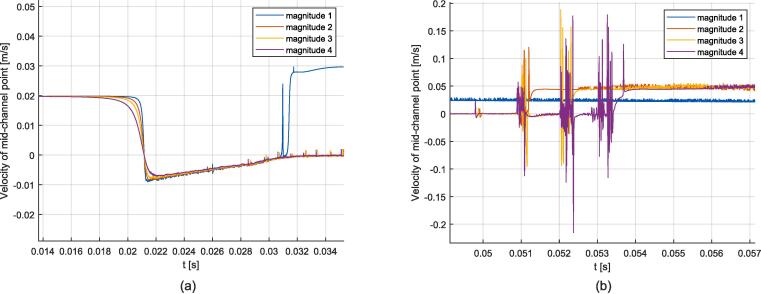
Evolution of the mid-channel point velocity (front of the advancing gas bubble) for the case of the advected gas bubble exposed to one-side resonance. (a) Start of the acoustic resonance. (b) End of acoustic resonance.

To deal with the case of a gas bubble advected towards a resonance position, the following configuration was considered: A flow velocity of 0.02 m/s was applied to the gas bubble starting from a position about 0.5 mm away from the resonance position, with the excitation located on the right boundary at four different magnitudes, see Table C.9. The evolution of the gas bubble shape for the case of magnitude 4 is illustrated in Fig. 15 and visualized in Video 11, additional videos are provided in the Supplementary Material, see reference in Appendix D. Figs. 16 (a) and 17 (a) depict the simulation results at the start of acoustic resonance, from 14 ms to 35 ms, and Figs. 16 (b) and 17 (b) plot the simulation results at the end of acoustic resonance, from 49 ms to 57 ms. Initially, an increase of the acoustic amplitude is observed, reaching values which are independent of the excitation magnitude and where flow and the acoustic force are balanced. For magnitude 1 the acoustic force is not strong enough to maintain this balanced regime, and the gas bubble returns to the advected equilibrium shape. For all other magnitudes resonance peaks are observed after the almost steady regime, *i.e.* one peak is observed for magnitude 2, two for magnitude 3, and three for magnitude 4. Then the bubble returns to the advected state.

### 3.6 Resonance shock on one side

**Fig. 18:**
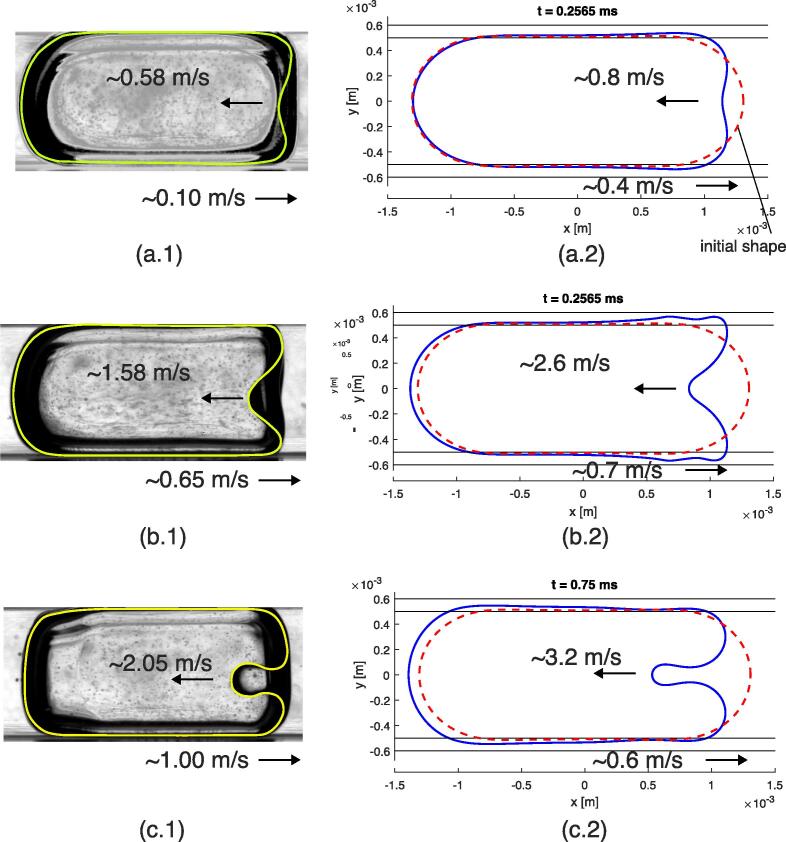
Qualitative comparison of maximum shape deformations for different resonance peak magnitudes. (a.1) and (a.2): Result for magnitude 1 compared with frame No. 14 of Video 5. (b.1) and (b.2): Result for magnitude 3 compared with frame No. 95 of Video 5. (c.1) and (c.2): Result for magnitude 5 compared with frame No. 36 of Video 5. Approximate instantaneous maximum velocities of inward and outward bubble deformation are indicated.

**Fig. 19:**
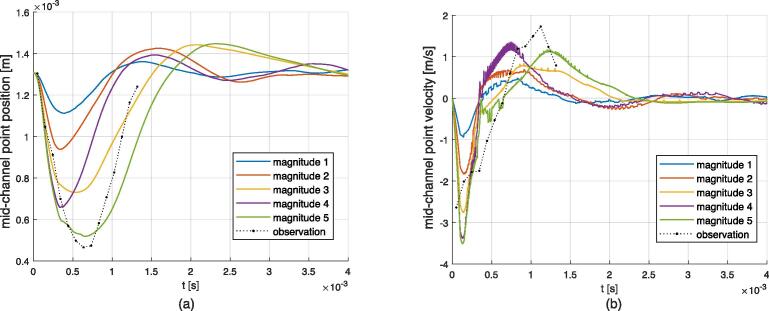
Result for different magnitudes of acoustic peak shock on a gas bubble. (a): Position of the mid-channel point of the sonicated face. (b): Velocity of the mid-channel point of the sonicated face. Observation is taken from Video 5 at shock happening at frame No. 36.

The obtained results highlight that an acoustic resonance peak can lead to a strong transient deformation of the gas–liquid interface when the acoustic force reaches a large magnitude for a short time. However, the value predicted by the acoustic solver may not be exact due to the singular nature of resonance. Therefore, it may be more rigorous to quantify acoustic resonance in terms of energy density of the associated peak, which would represent the product of the acoustic field amplitude by its extent in time. Using this description, a trivial resonance peak in the time-domain can be modeled as a rectangular pulse of period *T* and amplitude *A*, so that the energy density of the peak would be *T* × *A*. More generally, for a given time period *T*, it is possible to use the following mathematical definitions for the acoustic force energy density $E_{f}$:

(19) $$E_{f}=\int_{0}^{T}|f_{\mathrm{acoustic}}(t)|\mathrm{dt},$$

and for the pressure energy density $E_{p}$:

(20) $$E_{p}=\int_{0}^{T}|p_{1}(t)|\mathrm{dt},$$

where $f_{\mathrm{acoustic}}$ and $p_{1}$ are the maximum acoustic force and the acoustic pressure, respectively, computed by the acoustic solver. Simulation result are given for a 2.6 mm long gas bubble subjected to a strong resonance shock from equilibrium shape. The peak energy density values were selected according to the strongest deformations that were observed in practice. The values are given in Table C.10, and associated videos are available in the Supplementary Material, see reference in Appendix D. The single shock solution is also illustrated in Video 12 corresponding to the magnitude 3 case. The excitation consisted of a pulse of 1e-4 s duration, which corresponds to 1000 integration time steps and to 1 frame for a 10,000 fps video. Fig. 18 depicts the qualitative comparison of maximum shape deformations for different resonance peak magnitudes with experimental observations (Video 5). The evolution of both position and velocity of the mid-channel point for the five magnitudes is shown in Fig. 19. The shock produces a concave shape, whose extent depends on the magnitude. Traveling capillary waves are also observed along the interface.

**Fig. 20:**
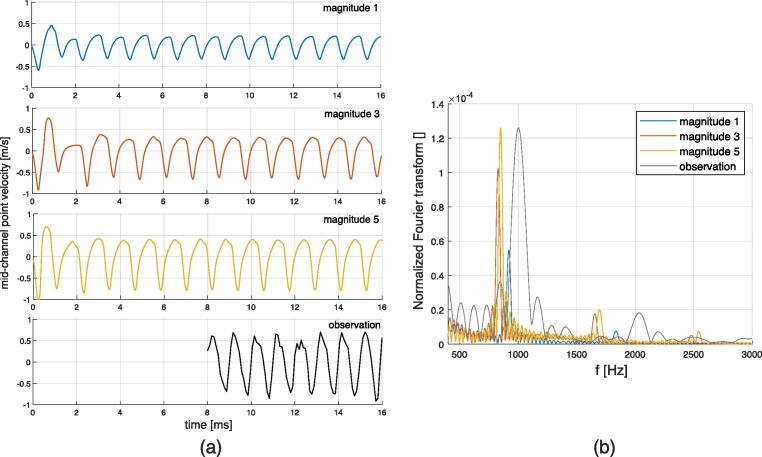
Results for the oscillating interface case at different magnitudes. (a): Mid-channel point velocity (negative velocity stands for displacement towards the inside of the gas bubble) for, from top to bottom, magnitude 1, magnitude 3, magnitude 5 and observation from Video 6. (b) Normalized Fourier transform for the signals in (a), (normalization according to the root-mean-square value of the signals).

When the gas bubble recovers its initial shape after the acoustic resonance shock, a new shock can arise, as shown in Video 6. According to this footage, standing Faraday waves emerge for about three frames, which corresponds to about 3e-4 s. In the following results, the excitation consisted of a sequence of pulses, equal in magnitude, of length 3e-4 s (sequence of square inputs). The pulse starts to be active when resonance is met, that is to say when a resonance peak is detected numerically. Simulations for different magnitudes of the resonance shock are performed, see Table C.11. Video 13 shows the periodic solution corresponding to the magnitude 3 case, and additional videos are available in the Supplementary Material, see reference in Appendix D. The mid-channel point oscillation is measured and plotted in Fig. 20 (a). After a transient stage of about 4 ms, the deformation of the interface follows a steady cycle. As for the previously discussed single shock results, velocities towards the inside (acoustic force pushing the interface) and the outside (surface tension recovering the shape) of the gas bubble are different. The measurement of the mid-channel point position in the experiment suffers from inaccuracies due to the difficulty to track the point in the footage. Consequently, the values of velocity are approximate. The Fourier spectrum of the oscillation for the different magnitudes is given in Fig. 20 (b). The measured oscillation frequency for Video 6 is about 1000 Hz, giving a period of 1 ms. Numerical results show that the oscillation frequency depends on the magnitude of the peak. Globally, one can observe that a stronger shock produces a more significant deformation that takes longer to recover, extending the oscillation period. It is expected that the recovery period depends on the static radius of curvature of the gas bubble, as the capillary pressure increases with decreasing radii of curvature. To confirm this, similar simulations were performed for two additional radii of curvature *R* of the gas bubble, varying slightly the channel height, for the case of magnitude 2. The oscillation frequencies for *R* = 0.5 mm (reference value), *R* = 0.45 mm, and *R* = 0.4 mm, were 918.4 Hz, 1226.1 Hz and 1365.2 Hz, respectively. The results of the oscillating gas bubble are summarized in Table 5.

**Table 5:** Simulation results for the oscillating gas bubble interface compared to experiments (Video 6). $|v_{2}{|}_{\mathrm{in}}$: maximum speed towards the inside of the gas bubble (acoustic force pushing the interface). $|v_{2}{|}_{\mathrm{out}}$: maximum speed towards the outside of the gas bubble (surface tension making the interface to recover). $f_{\mathrm{oscillation}}$: frequency of oscillations.

Configuration Ref.	$|v_{2}{|}_{\mathrm{in}}$	$|v_{2}{|}_{\mathrm{out}}$	$f_{\mathrm{oscillation}}$
Magnitude 1	0.34 m/s	0.21 m/s	920.9 Hz
Magnitude 2	0.45 m/s	0.27 m/s	918.4 Hz
Magnitude 3	0.68 m/s	0.32 m/s	828.0 Hz
Magnitude 4	0.74 m/s	0.37 m/s	835.4 Hz
Magnitude 5	0.79 m/s	0.43 m/s	850.0 Hz
Observation	0.91 m/s	0.70 m/s	1001.5 Hz

### 3.7 Faraday instability VOF results

**Fig. 21:**
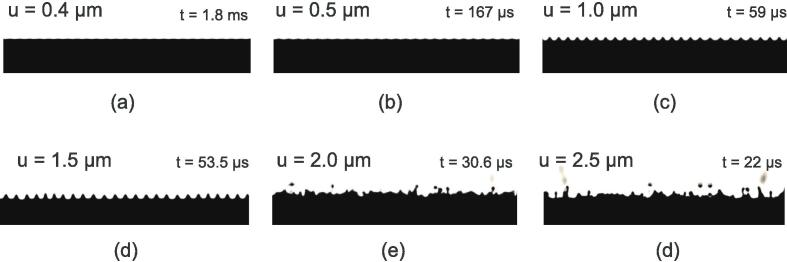
Contour plots of the phase fraction (extracted at α=0.5) at maximum of deformation of the free surface for the different simulations (water in black). The associated oscillation amplitude is indicated, and the time at which the image is taken is stated next to the image.

VOF simulations of the Faraday instability are presented in this section. The phase fraction (extracted at *α* = 0.5 ) at maximum deformation of the free surface for the six selected magnitudes is shown in Fig. 21. The complete videos of the simulated interface evolution are provided in the Supplementary Material, see reference in Appendix D. Small amplitude standing waves are observed for *u* = 0.4  μm and *u* = 0.5  μm. Typical crest-shaped standing wave starts to appear at *u* = 1.0  μm, which become higher at 1.5 μm. Chaotic motion and breaking are observed at *u* = 2.0  μm and *u* = 2.5 μm.

**Fig. 22:**
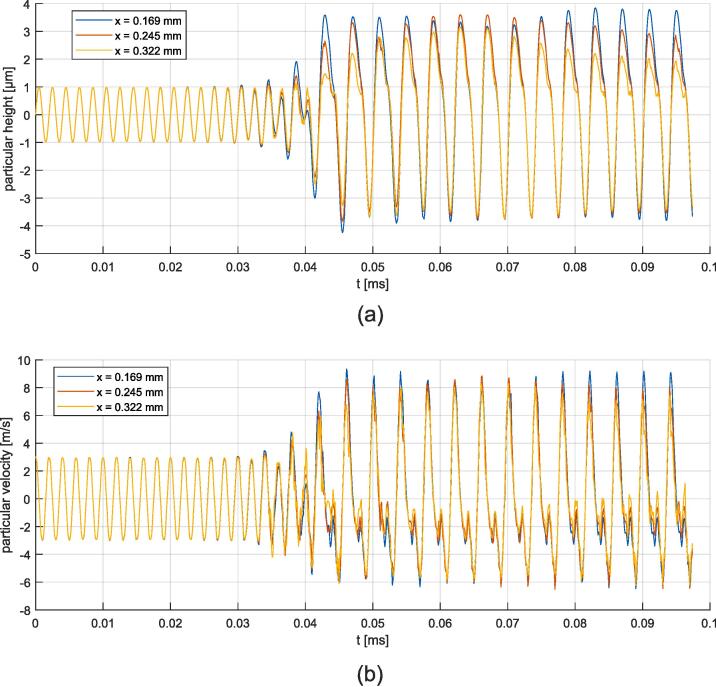
Time signals for the 1.0 μm forcing oscillation amplitude simulation at three different antinodes (whose abscissa is indicated in the legend box). (a) Particular height. (b) Particular vertical velocity.

**Fig. 23:**
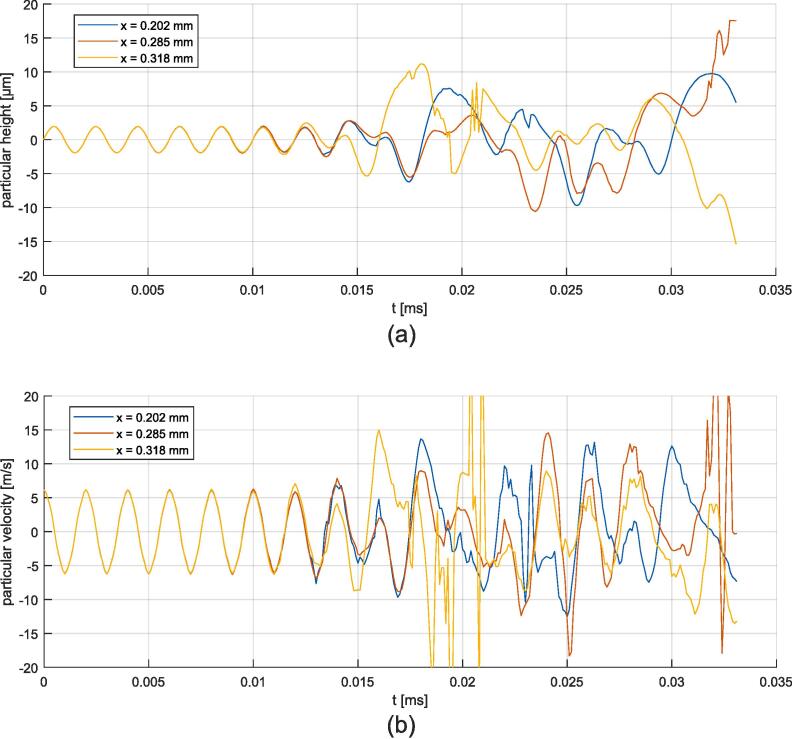
Time signals for the 2.0 μm forcing oscillation amplitude simulation at three different antinodes (whose abscissa is indicated in the legend box). (a): Particular height. (b): Particular velocity.

**Fig. 24:**
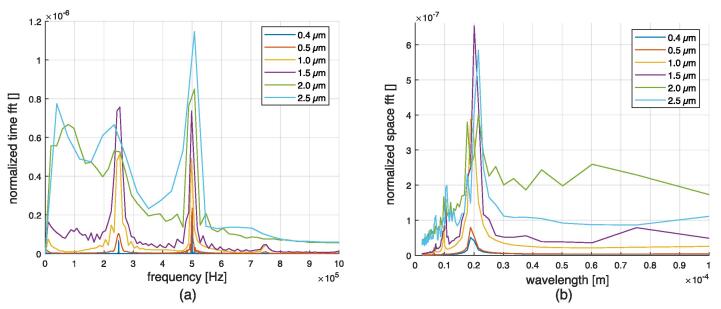
Synthetic Fourier analysis for the different amplitudes. (a): Space-averaging of the amplitude of time-fft. (b): Time-averaging of the amplitude of space-fft.

**Fig. 25:**
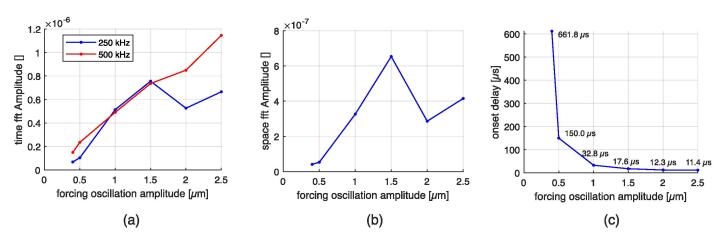
Quantities for the VOF simulation at the different forcing oscillation amplitudes. (a) Amplitude of ω/2/(2π) =  250 kHz and ω/(2π) =  500 kHz. (b) Amplitude of the peak at critical wavelength. (c) Onset time of the Faraday instability.

The temporal evolution of the surface height and its velocity is recorded at three points of the surface for magnitudes *u* = 1.0  μm and *u* = 2.0  μm, which are depicted in Fig. 22, Fig. 23, respectively. Velocity aberrations caused by bad interface capture are saturated in Fig. 23 (b). At the beginning of the simulation, the free surface oscillates homogeneously at the excitation amplitude. Then, the instability emerges, leading to periodic motion for u =  0.4, 0.5, 1.0 and 1.5 μm, or chaotic motion for u =  2.0 and 2.5 μm. The onset of the instability depends on the excitation amplitude. Fourier analysis of the surface motion is given in Fig. 24. The two synthetic quantities are the space-averaging of the amplitude of time-fft, and the time-averaging of the amplitude of space-fft. The amplitudes are normalized to facilitate a relative comparison. The synthetic time-fft highlights a peak at 500 kHz, corresponding to the forcing frequency, and a peak at 250 kHz, corresponding to the ω/2 subharmonic Faraday instability. The synthetic space-fft shows a peak at a wavelength of 0.2e-4 m, corresponding to the wavelength of the standing wave. Chaotic motion is demonstrated through widening of the spectrum for *u*  =  2.0 and 2.5 μm. Peak amplitudes and instability onset delay are plotted in Fig. 25. As expected, the 500 kHz peak follows an almost linear relation with respect to the forcing amplitude. The 250 kHz peak drops at 2.0 μm and 2.5 μm because of peak widening. The onset delay is very dependent on the forcing oscillation, with values from tens of *μ*s to hundreds of *μ*s for the smallest excitation amplitude.

## 4 Discussion

The selected simulation method correctly predicts the gas bubble flattening in a consistent manner with the experiments. The flattened shape is only obtained when updating the acoustic force with respect to the moving boundary, but not as a solution of the static problem. The most flattened shape is obtained for the advected case, for which the gas bubble is squeezed between the liquid slug and the acoustic standing wave, see *e.g.* Video 11 frame No. 2600. The comparison between free, constrained and advected configurations allows to reveal the dependence of the gas bubble behavior on the surrounding medium conditions. Since it is expected that the volume of the liquid slug between bubbles does not change, or only changes relatively slightly, the constrained bubble configuration may be more realistic than the free bubble configuration. However, it has to be noted that the thin liquid film between the gas bubble and the channel wall was not modeled, as it cannot be captured with the current modeling approach. Constrained and both-sides resonance configurations highlight a steady deformation state corresponding closely to what is observed in the microreactor. The gas bubble advected towards a resonance position exhibits a more complex behavior which may be encountered in practice. In this case, the resonance is met when the bubble reaches a particular position. Steady flattening is then obtained for a finite period, depending on the acoustic force magnitude.

Furthermore, both experiments and simulations demonstrate that there is a coexistence between the flattened shape solution and the shock solution. Both solutions can generate atomization at the deformed interface. We assert that the existence of a shock solution, which leads to transient concave deformation, is due to a short acoustic peak of high amplitude. The acoustic peak depends on the resonance conditions. Relatively good agreement is obtained for the concave deformation of the bubble, as shown in Fig. 18. For the case of strong deformation (*e.g.* see Fig. 18 (c.1) and (c.2)), it may be expected that the simplified Lagrangian Surface Evolver reaches its limits, since more incompressible flow effects occur near the strongly curved surface. Moreover, the standing wave approximation may reach its limits when dealing with such short acoustic excitation. A time-domain acoustic solver may be more relevant to deal with strong deformation that takes place for hundreds of microseconds. Notwithstanding, the dynamic behavior is well reproduced, but might be overestimated due to the overestimation of the acoustic force by the frequency-domain solver.

The predicted acoustic pressure is in the range of hundreds of kPa to tens of MPa. A large acoustic pressure manifests itself first with gas bubble flattening at speeds up to about 2 m/s, then atomization can emerge. Cavitation is determined directly by the acoustic pressure. Assuming that, at 500 kHz, cavitation arises likely around 100 kPa, the quantitative approach also predicts weak to strong cavitation in the liquid. This is consistent with the experimental observation of Mc Carogher et al. [27], where cavitation bubbles are generated and focused at the pressure nodes of the acoustic mode and at the gas bubble surface. In summary, the comparison of the experiments and the numerical results shows that a stable acoustic pressure value can go up to 2 MPa, and that atomization arises from 10 MPa. We propose that a resonance peak can lead to values up to 80 MPa, which we expect are then responsible for strong atomization and cavitation happening for tens of microseconds, as observed for example in Video 7.

VOF results allow to characterize atomization conditions for this precise configuration. The measured critical wavelength of about 20 μm (Fig. 24 (b)) is consistent with the K&T theory. This value can also be approximated by Kelvin’s formula $\lambda =2\pi {\left(4\gamma /(\rho_{0}\omega^{2})\right)}^{\left(1/3\right)}$, where ω is the forcing pulsation. Slightly above the theoretical critical oscillation amplitude of 0.35 μm, for the 0.4 μm simulation, a weak Faraday instability is observed. One can assert that for all the standing acoustic waves whose particular oscillation amplitude is below the K&T theoretical threshold, no standing Faraday wave can be observed. The associated acoustic pressure for the selected 500 kHz configuration is about 1.5 MPa. According to VOF results, it seems that the breakup threshold is between 1.5 μm and 2.0 μm. The associated acoustic pressure range would be from 6 to 10 MPa. Assuming pure (0,n) mode, one can use the formula relating the acoustic pressure and the horizontal oscillation amplitude: $p=u_{x}c_{0}\rho_{0}\omega$. This gives an acoustic pressure of 9.3 MPa for a 2.0 μm oscillation. One can compare those values with Goodridge’s equation for the selected constants [47], [48]:

(21) $$a_{d}\approx 0.26{\left(\frac{\gamma }{\rho_{0}}\right)}^{1/3}\omega^{4/3},$$

where $a_{d}$ is the critical driving acceleration of the onset of drop ejection. For 500 kHz, this formula gives a critical driving acceleration of 4.977e + 6 m/s^2^, thus an oscillation amplitude of 0.504 μm. Consequently, we found that this formula overestimates the atomization for the selected frequency. However, it has to be noted that this formula was validated in a frequency range from 25 Hz to 100 Hz in Puthenveettil and Hopfinger’s work [43], and therefore might not be directly applicable in the ultrasonic range. To obtain an atomization threshold of about 1.75 μm, corresponding to the one predicted in this study, the coefficient in Eq. (21) should be about 0.9 instead of 0.26. Results for the simplified VOF configuration showed that the interface vibration pattern of mode (0,n) is quite homogeneous around the mid-channel point, as depicted in Fig. 9 first row second column. One can expect that more complex atomization patterns are associated with more complex vibration patterns, which may have nodes along the channel height.

Assuming that an acoustic pressure greater than 10 MPa is required to generate a sufficiently observable atomization, the numerical results demonstrate that atomization only happens during strong transient deformations associated with resonance. The simulated steady states involve steady acoustic pressures below 3 MPa, which is only sufficient to produce at most a standing Faraday wave. This assertion is consistent with the experiments, see *e.g.* Video 5 frame No. 182 and Video 7 frame 288. Most of atomization appears as what one can call *atomization bursts*. Thus, we found that a possible steady atomization behavior would consist of a sequence of atomization bursts. This can happen in both the flattened and shock case. The shock solution, for the constrained gas bubble, can be periodic, since the gas bubble can recover its initial shape. For the oscillating solution, the dynamic behavior is well reproduced, as a similar oscillating shape is obtained. The predicted oscillation frequency is from 850 to 920 Hz, compared to an experimental result of 1000 Hz (from Video 6). The recovery time is determined inherently by the surface tension to density ratio, but also by the magnitude of the acoustic force. Little adjustments of these parameters may give a better fit of the oscillation frequency. But given the simplicity of the assumptions leading to these results, we propose that the oscillation frequency discrepancy is acceptable. Furthermore, in practice, due to the fact that the acoustic resonance is highly sensitive to geometry changes, a perfectly periodic behavior is not observed. The typical recovery period obtained by the simulations can take values from 1 to 5 ms, see Fig. 19 (a), so irregular oscillations with variable period in this range can be expected.

## 5 Conclusions and perspectives

A numerical approach combining an acoustic frequency-domain solver and a Lagrangian Surface-Evolver solver was developed to describe the acoustic deformation of gas–liquid interfaces. The method was designed to describe phenomena driven by dynamic acoustic resonance. In such problems, nonlinearity and high dynamic range are expected. We focused on the acoustic resonance happening between gas bubbles in a segmented two-phase flow in a microchannel sonicated at 500 kHz. Globally, the behavior of the bubble submitted to acoustic resonance is well reproduced, with good qualitative agreement with experiment and consistent order of magnitude. The direct relation between acoustic field and interface deformation was established. We concluded that the coexistence of steady flattening and strong transient concave deformation of gas bubbles is consistent with selected hypotheses. We found that the acoustic pressure magnitude can take values from hundreds of kPa to tens of MPa. These values are consistent with the observation of atomization and cavitation in the experiments. VOF simulations allowed to highlight atomization generation conditions and were put into perspective with acoustic resonance. We found that dynamic acoustic resonance gives rise to atomization bursts at the gas bubble surface.

In the presented developments, we focused on a simplified configuration of the microreactor and we considered vibration homogeneous in space along the microreactor plane. The presented approach can be applied to more complex acoustic fields involving more complex channel geometry, the actual vibration pattern of the ultrasound transducer, or more complex two-phase flow. The method can also be extended to 3D to model Faraday crystals and the actual atomization pattern. For the selected configuration, the 3D extension would have led to similar conclusions regarding gas bubble deformation. The selected numerical framework allows not only to predict the phenomena numerically, but can also be used in inverse problems to characterize the design of ultrasonic microreactors.

## CRediT authorship contribution statement

**William Cailly:** Conceptualization, Methodology, Investigation, Writing - original draft. **Keiran Mc Carogher:** Methodology, Investigation, Writing - original draft. **Holger Bolze:** Investigation, Writing - review & editing. **Jun Yin:** Investigation, Writing - review & editing. **Simon Kuhn:** Funding acquisition, Supervision, Writing - review & editing.

## Declaration of Competing Interest

The authors declare that they have no known competing financial interests or personal relationships that could have appeared to influence the work reported in this paper.

## Figures and Tables

**Table C.6:** Input values for the one-side transient flattening on free gas bubble simulations.

Configuration Ref.	excitation velocity	excitation amplitude	frequency
Magnitude 1	0.018 m/s	0.573e−8 m	500 kHz
Magnitude 2	0.036 m/s	1.150e−8 m	500 kHz
Magnitude 3	0.072 m/s	2.292e−8 m	500 kHz
Magnitude 4	0.108 m/s	3.438e−8 m	500 kHz
Magnitude 5	0.126 m/s	4.011e−8 m	500 kHz

**Table C.7:** Input values for one-side transient flattening simulations on constrained gas bubble simulations.

Configuration Ref.	excitation velocity	excitation amplitude	frequency
Magnitude 1	0.04 m/s	1.27e−8 m	500 kHz
Magnitude 2	0.06 m/s	1.91e−8 m	500 kHz
Magnitude 3	0.08 m/s	2.55e−8 m	500 kHz
Magnitude 4	0.10 m/s	3.18e−8 m	500 kHz
Magnitude 5	0.12 m/s	3.82e−8 m	500 kHz
Magnitude 6	0.14 m/s	4.46e−8 m	500 kHz
Magnitude 7	0.16 m/s	5.09e−8 m	500 kHz

**Table C.8:** Input values for both-sides resonance with bottom excitation simulations.

Configuration Ref.	excitation velocity	excitation amplitude	frequency
Magnitude 1	0.0942 m/s	3.0e−8 m	500 kHz
Magnitude 2	0.1257 m/s	4.0e−8 m	500 kHz
Magnitude 3	0.1916 m/s	6.1e−8 m	500 kHz
Magnitude 4	0.3142 m/s	10.0e−8 m	500 kHz

**Table C.9:** Input values for advected gas bubble simulations.

Configuration Ref.	excitation velocity	excitation amplitude	frequency
Magnitude 1	8e−3 m/s	2.55e−9 m	500 kHz
Magnitude 2	16e−3 m/s	5.09e−9 m	500 kHz
Magnitude 3	24e−3 m/s	7.64e−9 m	500 kHz
Magnitude 4	40e−3 m/s	12.73e−9 m	500 kHz

**Table C.10:** Input values for one-side resonance shock simulations. The energy densities $E_{f}$ and $E_{p}$ are associated with a single peak.

Configuration Ref.	peak force energy $E_{f}$	peak pressure energy $E_{p}$	frequency
Magnitude 1	1.00e3 Pa ⋅s ⋅m^−1^	433 Pa ⋅s	500 kHz
Magnitude 2	1.75e3 Pa ⋅s ⋅m^−1^	573 Pa ⋅s	500 kHz
Magnitude 3	2.50e3 Pa ⋅s ⋅m^−1^	684 Pa ⋅s	500 kHz
Magnitude 4	3.00e3 Pa ⋅s ⋅m^−1^	750 Pa ⋅s	500 kHz
Magnitude 5	3.10e3 Pa ⋅s ⋅m^−1^	762 Pa ⋅s	500 kHz

**Table C.11:** Input values for one-side resonance oscillating interface simulations. The energy densities $E_{f}$ and $E_{p}$ are associated with a single peak. peak |p|: equivalent magnitude of acoustic pressure during the resonance peak, peak |u|: equivalent magnitude of acoustic oscillation amplitude during the resonance peak.

Configuration Ref.	peak pressure energy $E_{p}$	peak length	peak |p|	peak |u|
Magnitude 1	374 Pa⋅ s	3e−4 s	1.25 MPa	0.39 μm
Magnitude 2	433 Pa ⋅s	3e−4 s	1.44 MPa	0.45 μm
Magnitude 3	484 Pa ⋅s	3e−4 s	1.62 MPa	0.68 μm
Magnitude 4	530 Pa ⋅s	3e−4 s	1.77 MPa	0.74 μm
Magnitude 5	572 Pa ⋅s	3e−4 s	1.91 MPa	0.79 μm

**Table D.12:** List of supplementary videos.

Folder	Content			
AdvectedBubble	One-side resonance on an advected gas bubble,			
	4 magnitudes			
BothSidesResonance	Both-sides resonance, 4 magnitudes			
ConstrainedBubble	One-side transient flattening on constrained bubble,			
	7 magnitudes			
FaradayVOFopenfoam	Faraday instability VOF results, 5 magnitudes			
FreeBubble	One-side transient flattening on free bubble,			
	5 magnitudes			
OscillatingBubble	Oscillating bubble on one side, 5 magnitudes			
Shock	Resonance shock on one side, 5 magnitudes			
SurfaceEvolverCodeBenchmarks	Simulations for Surface Evolver code validation			

## Appendix B Analytical solution: case of the steady deformation of a liquid film

An analytical solution can be found for the equations describing the surface deformation by the acoustic radiation force. Especially, one can consider the case of the steady deformation of an infinitely long two-dimensional liquid film of height *H*. Let us place the upper free surface at y=H and the fixed bottom face at y=0. Let the boundary conditions for the first order acoustic equation be $p_{1}=0$ (free surface) at y=H and $v_{y,1}=0$ at y=0 (non-penetration and slip condition). It can be shown that when taking γ =  0.072 N/m the acoustic pressure is negligible and is about 1e-7 times the maximum acoustic pressure, which justifies in most cases the Dirichlet boundary condition on a free surface for the acoustic wave computation. A pure harmonic solution of the linear standing acoustic wave can have the form:

(B.1) $$p_{1}(x,y,t)=p_{a}\cos(k_{y}y)\cos(k_{x}x)\sin(\omega t),$$

(B.2) $$v_{y,1}(x,y,t)=-v_{a}\sin(k_{y}y)\cos(k_{x}x)\cos(\omega t),$$

with

(B.3) $$k_{y}=\frac{(2n-1)\pi }{2H},n\in \{1,2,3,\ldots \},$$

and

(B.4) $$k_{x}^{2}+k_{y}^{2}=\frac{\omega^{2}}{c_{0}^{2}},$$

and where $p_{a}$ is the amplitude of the acoustic pressure, $v_{a}$ is the amplitude of the vertical acoustic oscillation velocity. The relation $v_{a}=k_{y}p_{a}/(\rho_{0}\omega )$ holds.

One can demonstrate by applying both Navier–Stokes and Zakharov approaches, described in Section 2.4, with associated assumptions, that the steady ($\partial_{t}\propto 0$) normal displacement of the upper surface at y=H is:

(B.5) $$\eta_{2,\mathrm{steady}}(x,y=H)=\frac{\rho_{0}v_{a}^{2}}{32k_{x}^{2}\gamma }\cos(2k_{x}x)\text{.}$$

It is possible to define the acoustic Weber number ${\mathrm{We}}_{\mathrm{acoustic}}$ as:

(B.6) $${\mathrm{We}}_{\mathrm{acoustic}}=\frac{\rho_{0}v_{a}^{2}L}{\gamma }\text{.}$$

From Eq. (B.5), the characteristic length *L* in the case of the liquid film is the wavelength in the *x* direction. The acoustic deformation of fluids can be described synthetically by ${\mathrm{We}}_{\mathrm{acoustic}}$. In the presence of dynamic acoustic resonance, ${\mathrm{We}}_{\mathrm{acoustic}}$ can vary greatly because of the large dynamic range of acoustic magnitude.

## Appendix D Supplementary material

The supplementary material consists of a set of videos. Videos referred Video N in the text are directly available in the electronic version of the article. Other videos are given in a zip file and grouped into different folders described in Table D.12. The excel sheet named VideosListInZip.xlsx, given in the same zip file, contains the description of each video.

## Supplementary data

Supplementary data 13

Supplementary data 14 Videos in Zip.

Supplementary data 15 Video details list

Supplementary data 16 Time step.

## Footnotes

1 acoustic force in short form.

2 convention used: $F(k)=\int_{-\infty }^{\infty }f(x)e^{-\mathrm{ikx}}\mathrm{dx}$
